# Arterial bullet embolism after thoracic gunshot wound

**DOI:** 10.1590/1677-5449.005315

**Published:** 2018

**Authors:** Raquel Magalhães Pereira, José Emerson dos Santos Souza, Antônio Oliveira de Araújo, Priscilla Ribeiro dos Santos, Ricardo Dias da Rocha, Marcos Henrique Parisati, Marcos Velludo Bernardes, Leonardo Pessoa Cavalcante

**Affiliations:** 1 Universidade Federal do Amazonas – UFAM, Hospital Universitário Getúlio Vargas – HUGV, Manaus, AM, Brasil.; 2 Universidade Federal do Amazonas – UFAM, Hospital Universítário Francisca Mendes – HUFM, Manaus, AM, Brasil.; 3 Universidade Federal do Amazonas – UFAM, Faculdade de Medicina, Manaus, AM, Brasil.

**Keywords:** embolism, wounds gunshot, aorta, femoral artery, endovascular procedures, embolia, ferimentos por arma de fogo, aorta, artéria femoral, procedimentos endovasculares

## Abstract

Bullet embolization of the arterial or venous systems is a rare complication of penetrating gunshot injuries. A 29-year-old man presented at the emergency department with a gunshot wound to the left arm, which had transfixed the arm and entered the thorax, with no exit wound. Initial radiographies showed a projectile in the upper left thigh. Contrast-enhanced tomography showed a pseudo-aneurysm of the descending thoracic aorta and the bullet inside the proximal left superficial femoral artery. Physical examination found diminished left pedal pulses, and the patient complained of left toe numbness. Endovascular thoracic aortic pseudoaneurysm repair was performed, sealing the descending aortic orifice with an endograft, and thromboembolectomy/bullet retrieval was carried out via a left femoral incision, both successfully. Considering that diagnosis of missile emboli depends on a high degree of suspicion, physicians who manage gunshot wound patients must be acutely aware of the possibility of intravascular bullet embolism.

## Resumo

A embolia balística pelo sistema arterial ou venoso é uma complicação rara de ferimentos penetrantes por arma de fogo. Um homem de 29 anos se apresentou na emergência com um ferimento por arma de fogo no braço esquerdo, que transfixou o braço e atingiu o tórax, sem ferimento de saída. Radiografias iniciais mostraram o projétil na coxa superior esquerda. A tomografia contrastada mostrou um pseudoaneurisma da aorta torácica descendente e o projétil no interior da artéria femoral superficial proximal esquerda. Ao exame físico, o pulso pedioso esquerdo estava diminuído e o paciente referiu dormência no hálux esquerdo. Foi realizado o reparo endovascular da aorta torácica e a tromboembolectomia/retirada do projétil por incisão femoral esquerda, ambos bem-sucedidos. Considerando que o diagnóstico de embolia balística depende de um alto grau de suspeição, os médicos que manejam pacientes com ferimentos por arma de fogo devem estar atentos a essa possibilidade.

## INTRODUCTION

 Intravascular bullet embolization is a rare phenomenon with potentially devastating consequences. Projectiles have been reported to migrate within arterial and venous circulation, resulting in life-threatening injuries from the initial injury as well as from the migration event. 1 ^,^ 2

 Due to their rarity, bullet embolisms are responsible for a considerable amount of diagnostic confusion. Careful evaluation of the projectile trajectory is therefore essential to identify all related injuries. 3

 Treatment for these conditions is decided on a case-by-case basis and may or may not involve removal of the projectile. 4 ^,^ 5 We present a case report of an arterial embolism treated with both endovascular and open surgical approaches. 

## CASE DESCRIPTION

**Figure 1:**
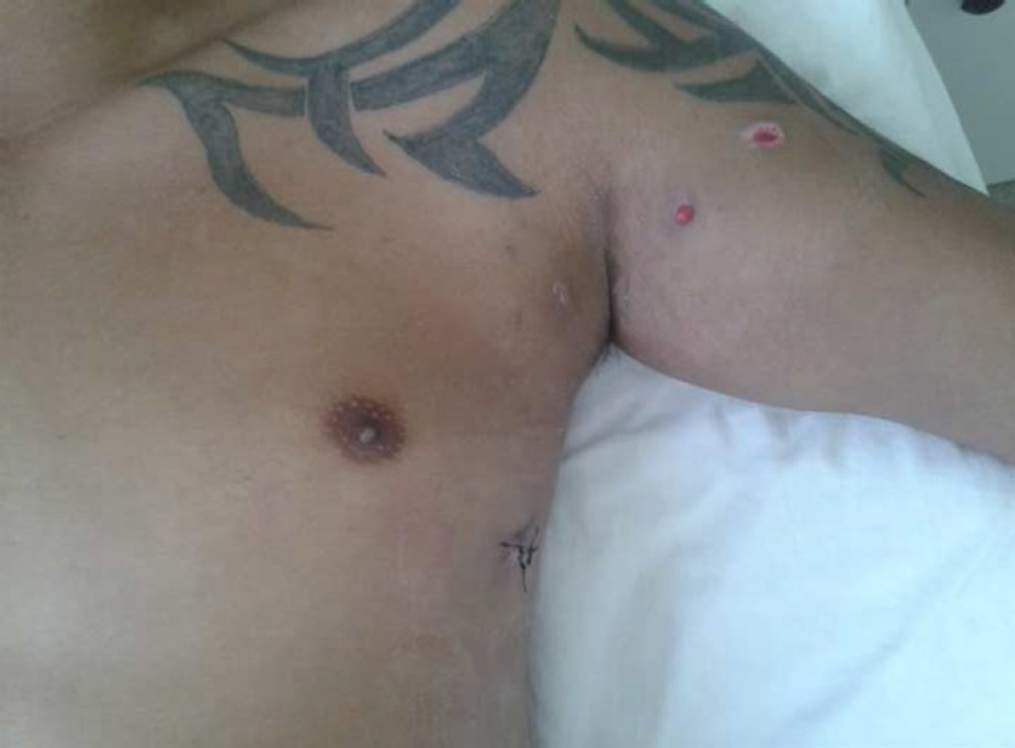
Gunshot wound to the left arm that transfixed the anterior area of the arm and entered the thorax.

 A 29-year-old man presented at the emergency department with a gunshot wound to the left arm that had transfixed the anterior area of the arm (near the shoulder) and hit the thorax in the axillary area ( Figure 1 ). There was no exit wound. 

 The patient was hemodynamically stable, but exhibited dyspnea and decreased breath sounds from the left chest. The remainder of the physical examination was unremarkable. The left thorax was drained through a chest tube, and the patient was then transferred to a reference trauma hospital. 

**Figure 2:**
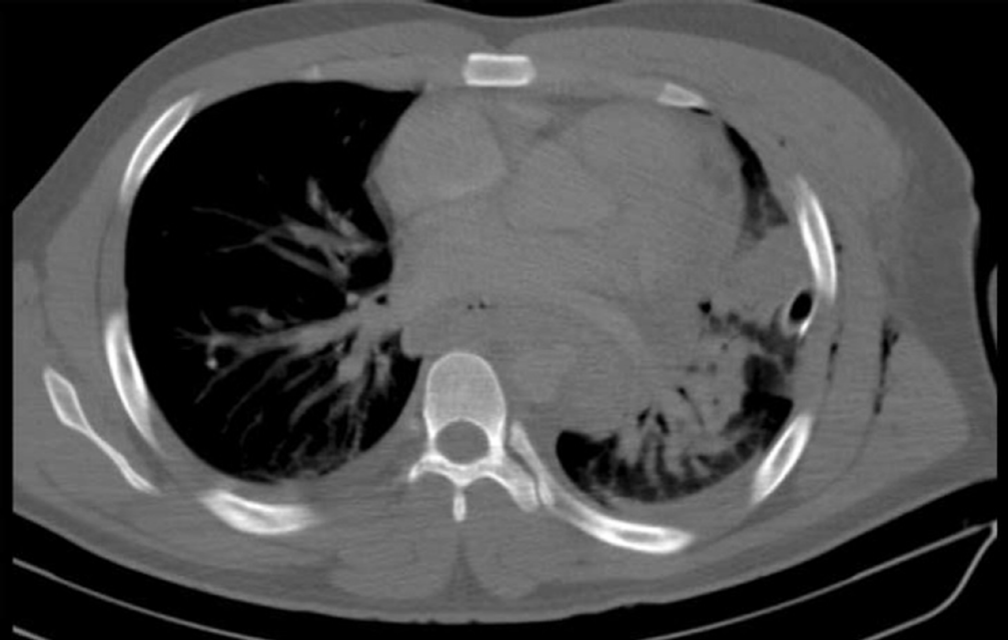
Contrast-enhanced tomography showing a pseudoaneurysm in the descending thoracic aorta.

**Figure 3:**
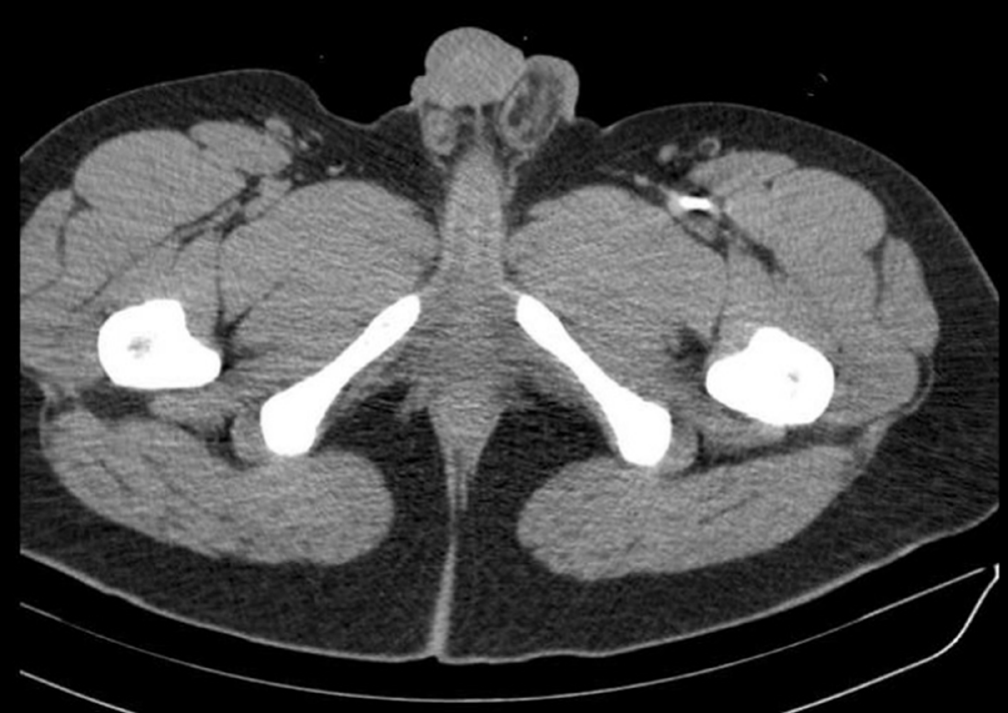
Contrast-enhanced tomography showing the bullet inside the proximal superficial femoral artery.

 The initial radiographic examination showed a projectile in the upper left thigh. Contrast-enhanced tomography showed a pseudo-aneurysm in the descending thoracic aorta ( Figure 2 ) and located the bullet inside the proximal superficial femoral artery ( Figure 3 ). The secondary physical examination found diminished left pedal pulses, with no temperature change in comparison to the contralateral limb, and the patient experienced left toe numbness. 

**Figure 4:**
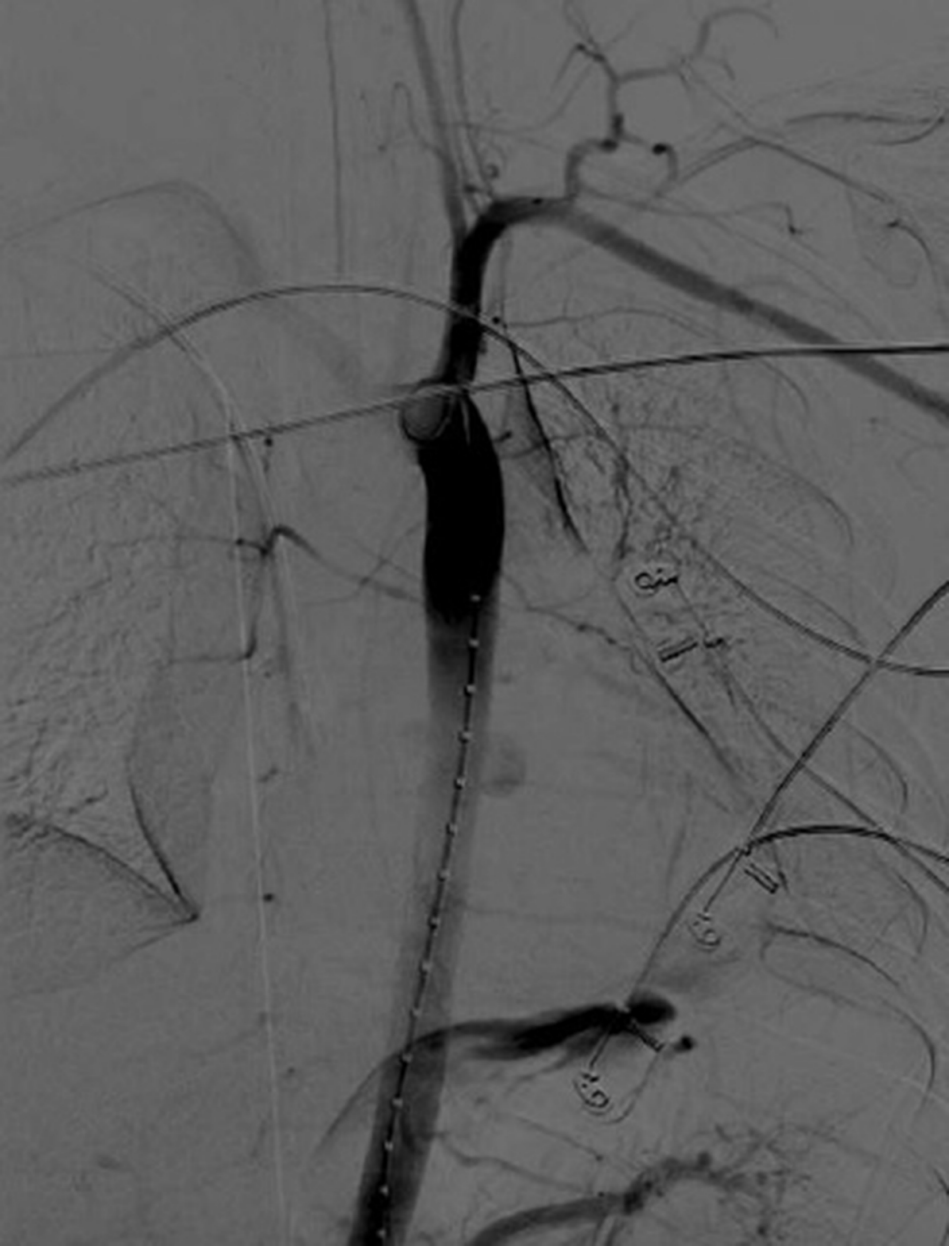
Pigtail angiographic control after the initial right femoral puncture, showing the pseudoaneurysm in the descending thoracic aorta.

**Figure 5:**
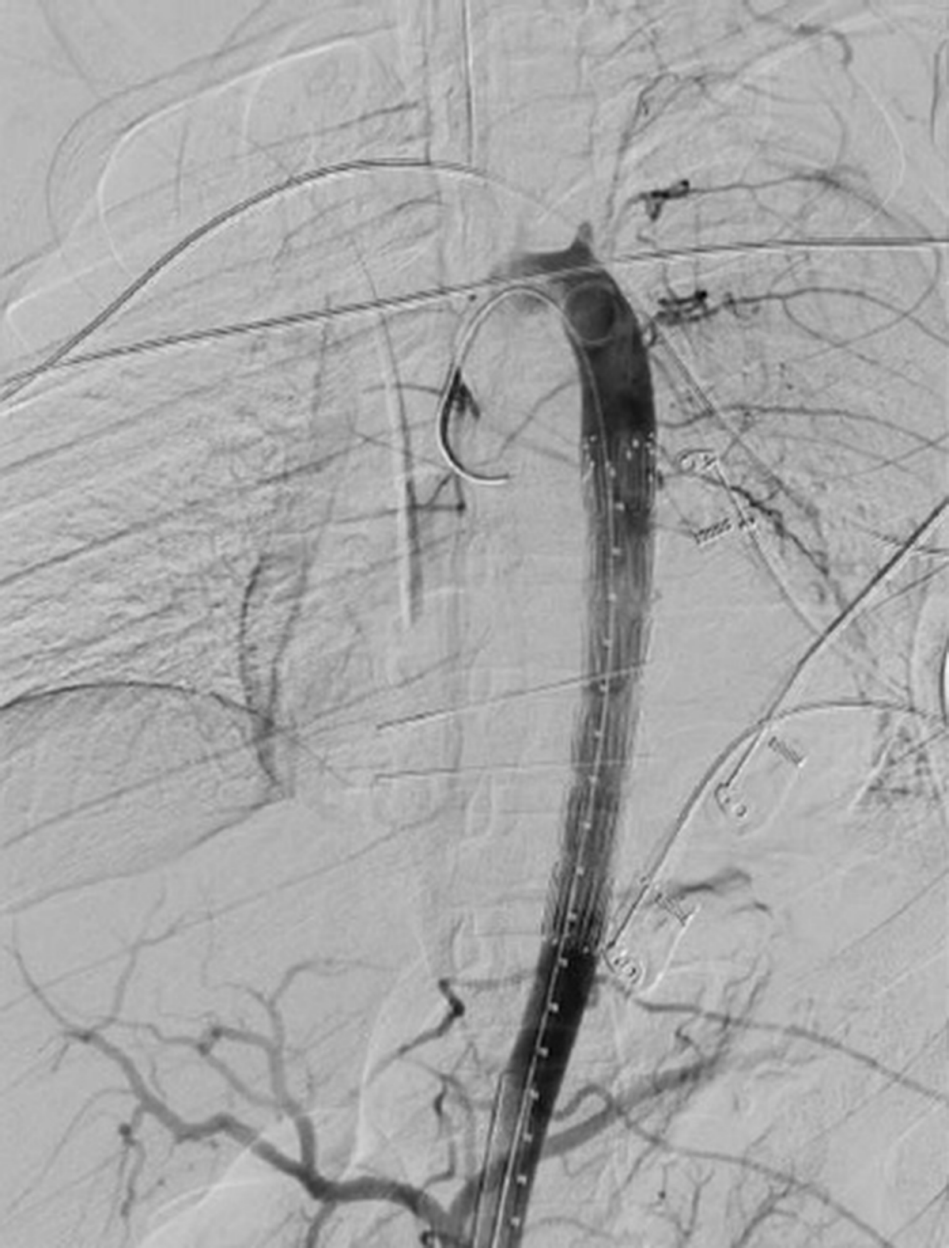
Retrograde positioning of a 24 mm × 130mm endograft that adequately sealed the descending thoracic aortic defect.

**Figure 6:**
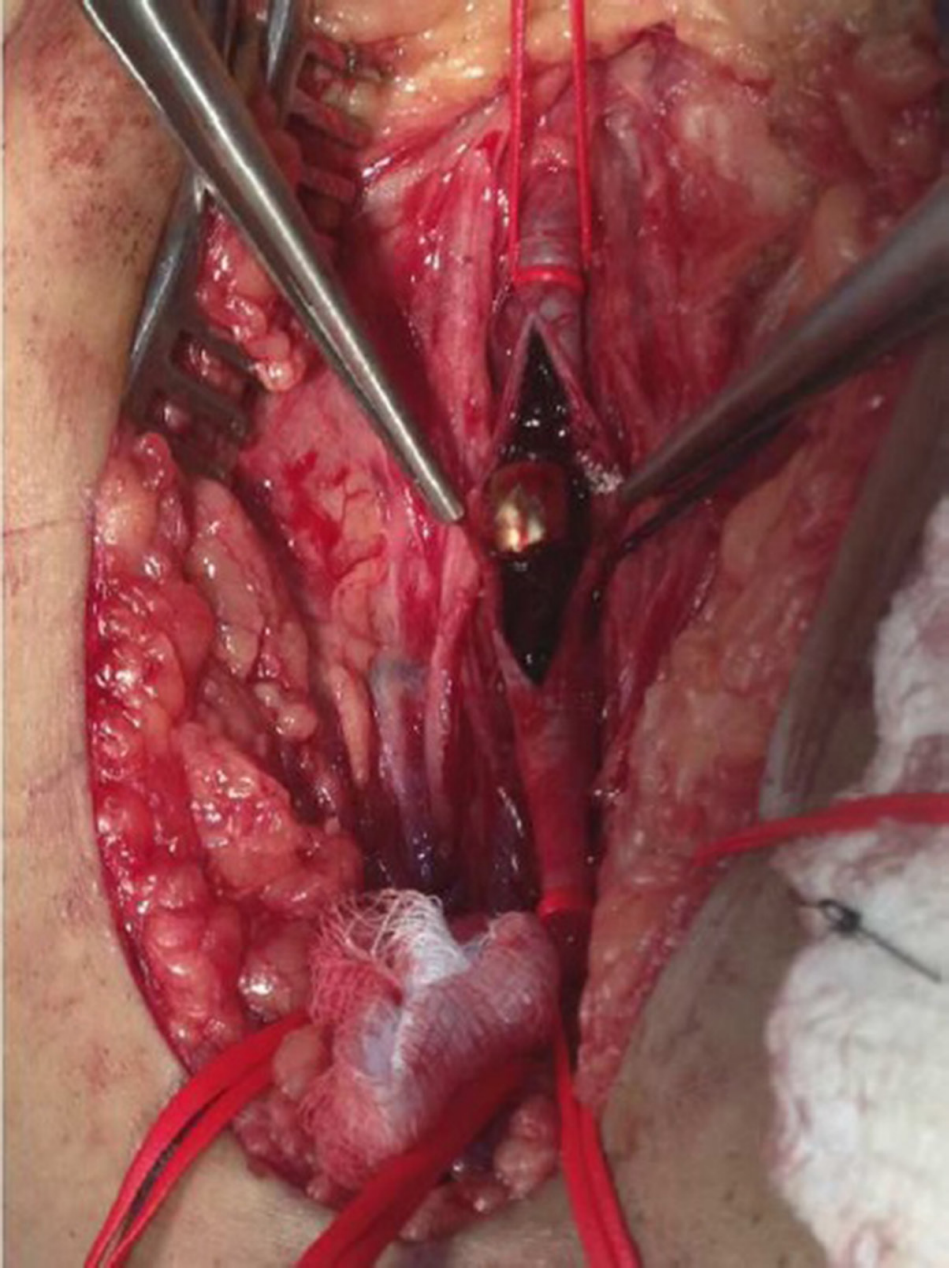
Bullet retrieval/thromboembolectomy after a left femoral incision.

 The patient was then transferred to a hybrid operation room and, after initial right femoral puncture and pigtail angiographic control ( Figure 4 ), a left femoral incision was made followed by retrograde positioning of a 24 mm × 130 mm endograft that adequately sealed the descending thoracic aortic defect ( Figure 5 ) and allowed for bullet retrieval and thromboembolectomy ( Figure 6 ). The patient had an uneventful recovery and was discharged on postoperative day 5. 

## DISCUSSION

 Generally, a bullet that penetrates the human body travels in a straight line and either goes through it, leaving the body or dissipates its kinetic energy and stops inside soft tissues, in which case the bullet can generally be found on radiological examination. 

 However, low-speed and/or low-energy projectiles may not travel in a straight line and may not exit the body. Additionally, they may have sufficient energy to pierce the wall of an arterial or venous vessel, but not enough energy to exit the vessel through another hole, thereby entering the vascular system. 6 ^,^ 7 This event is a rare complication of gunshot injuries and its occurrence was documented in 0.3% of 7500 casualties in the Vietnam War. 7 ^,^ 8

 The majority of cases involve the arterial system with antegrade migration, as in the case described here. There are fewer reports of venous involvement, which accounts for 20-25% of cases. 1 ^,^ 7 ^,^ 9

 According to a literature review, 10 from the first case of a projectile embolus, reported in 1834, up to 1990, approximately 160 cases had been reported. 2 Morais Filho et al. 5 found a total of 20 reported cases of arterial embolism since 1990, involving 23 occluded arteries. The arteries most often involved in these embolisms were the lower limb arteries (11 out of 22 cases, 50%). In 1968, Trimble 11 also reported similar data in a review of 33 cases of arterial bullet emboli following gunshot injury to the thorax. Specifically, the lower limb arteries (iliac, femoral, popliteal, and posterior tibial) accounted for 24 cases (72%), versus only 7 cases involving embolization to the upper extremities. 

 The site of impaction depends on the projectile caliber and vessel diameter and mainly occurs at vascular bifurcations, because they have sudden reductions in caliber. 1 ^,^ 2 ^,^ 4 Other contributory factors may include the patient's position following wounding, respiratory and hemodynamic activity, and gravity. 11 ^,^ 12 The vessel most commonly occluded in arterial bullet embolism is the femoral artery (30 to 50% of cases). 2 ^,^ 4 Additionally, the left leg is more commonly involved than the right, because of the smaller angle between the left common iliac artery and the aorta. 11 ^,^ 13 ^,^ 14

 In this case, the bullet probably penetrated the thoracic aorta, resulting in a pseudoaneurysm, and underwent antegrade migration to the left femoral bifurcation, occluding the proximal superficial femoral artery. 

 A bullet embolus should be suspected in the absence of an exit wound, 2 ^,^ 12 when there is radiological evidence of the presence of a bullet in unusual anatomic sites, without any relationship to the entrance wound, and/or where radiographic evidence does not correspond to the expected bullet trajectory. 2 ^,^ 4 ^,^ 6 Migration usually occurs soon after the missile enters the circulation, but it can also occur days, weeks, or even years later. 3 ^,^ 4 ^,^ 6

 Patients are asymptomatic in 70% of cases, especially when embolism is venous. Symptoms, when present, depend on the location of the bullet and its complications, such as thrombosis, arrhythmias, valve dysfunction, endocarditis, sepsis, erosion, or vascular occlusion. 2 ^,^ 4 In this case, the patient was relatively asymptomatic and the only manifestations were diminished left pedal pulses and a complaint of left toe numbness, with no temperature change in comparison to the contralateral limb. However, with a bullet impacted on the left femoral superficial artery, other signs and symptoms of limb ischemia would probably have appeared, if thromboembolectomy had not been performed promptly. 

 When bullet embolization is suspected, the management algorithms will depend on hemodynamic status and will usually begin with a CT scan and angiography, if the patient’s clinical condition permits. 15 Percutaneous extraction should then be considered, if feasible. Emergency surgical exploration will often be necessary, especially in arterial embolization cases. 1

 There is little consensus on treatment and indications for removal of bullet emboli. 6 ^,^ 12 However, most authors recommend conservative treatment in asymptomatic patients and recommend surgical removal of the bullet when there are symptoms or if there is a possibility of complications from leaving it in place. 2 ^-^ 4 ^,^ 7 In the absence of data to guide the decision to remove or observe fragment emboli, clinical common sense plays an important role and would support the observation of small asymptomatic emboli. 16

 The choice of surgical procedure is based on the exact location of the projectile and the patient’s clinical condition. The endovascular approach has been considered as the first treatment option for mobile projectiles, with percutaneous removal using basket or snare type catheters being the most frequently used technique. 1 ^,^ 4

 In general, in cases involving the presence of a thrombus, impaction of the bullet, and/or acute risks, an open approach must be considered for performing bullet retrieval/thromboembolectomy, as in this case. This is to avoid real dangers that will exist, as long as the missile is left within the circulation. These include further missile migrations and distal impactions, with consequent organ or limb infarction, thrombus propagation, erosion of vessels, and/or penetration into the adjacent structures, septicemia, lead intoxication, and death. 10

 The additional need for an endograft implant also contributed to the open surgery approach/access in this case. Thus, retrieval of the bullet and repair of the aortic pseudoaneurysm (introduction of the endograft delivery system) could both be performed through the same surgical incision. 

 Considering that diagnosis of missile emboli depends on a high degree of suspicion, physicians who manage gunshot wound patients must be acutely aware of the possibility of intravascular bullet embolism. Radiological exams are extremely important to determine the position of the projectile and guide the appropriate treatment, which must also always take into consideration the patient’s clinical condition. 

## Erratum

The article “Arterial bullet embolism after thoracic gunshot wound”, first published ahead of print on Aug 30, 2018 and finally in Jornal Vascular Brasileiro 17(3):262-266, DOI: https://doi.org/10.1590/1677-5449.005315 ; was published with the following sections missing, due to desktop publishing error:

- Correspondence,
- Author Information, and
- Author Contributions.

The missing content that should have been published may be found below:

**Correspondence ** Leonardo Pessoa Cavalcante Hospital Universitário Francisca Mendes, Serviço de Cirurgia Vascular e Endovascular Av. Camapuã, 108 - Cidade Nova II CEP 69097-720 - Manaus (AM), Brazil Tel.: +55 (92) 3649-2750 / +55 (92) 3649-2839 E-mail: leonardocavalcante@usp.br

**Author information ** RMP - General surgeon, Programa de Residência Médica em Cirurgia Geral, Hospital Universitário Getúlio Vargas, Universidade Federal do Amazonas (UFAM). JESS - Vascular surgeon, Serviço de Cirurgia Vascular e Endovascular, Hospital Universitário Francisca Mendes, Universidade Federal do Amazonas (UFAM). AOA - Vascular surgeon, Programa de Residência Médica em Cirurgia Vascular, Hospital Universitário Getúlio Vargas, Hospital Universitário Francisca Mendes, Universidade Federal do Amazonas (UFAM); Graduate (Professional Master’s), Programa de Pós-graduação em Cirurgia, Faculdade de Medicina, Universidade Federal do Amazonas (UFAM). PRS - Vascular surgeon, Programa de Residência Médica em Cirurgia Vascular e Endovascular, Hospital Universitário Getúlio Vargas, Hospital Universitário Francisca Mendes, Universidade Federal do Amazonas (UFAM). RDR - Vascular surgeon, Serviço de Cirurgia Vascular, Hospital Universitário Francisca Mendes, Universidade Federal do Amazonas (UFAM). MHP - Vascular surgeon, Programa de Residência Médica em Cirurgia Vascular, Hospital Universitário Getúlio Vargas, Hospital Universitário Francisca Mendes, Universidade Federal do Amazonas (UFAM). MVB - Vascular surgeon, Serviço de Cirurgia Vascular e Endovascular, Hospital Universitário Francisca Mendes, Universidade Federal do Amazonas (UFAM); Professor, Faculdade de Medicina, Universidade Federal do Amazonas (UFAM); Graduate (Professional Master’s), Programa de Pós-graduação em Cirurgia, Faculdade de Medicina, Universidade Federal do Amazonas (UFAM). LPC - PhD in Sciences (Medicine), Universidade de São Paulo; Vascular surgeon, Serviço de Cirurgia Vascular e Endovascular, Hospital Universitário Francisca Mendes, Universidade Federal do Amazonas (UFAM); Professor, Programa de Pós-graduação em Cirurgia, Faculdade de Medicina, Universidade Federal do Amazonas (UFAM).

**Author contributions ** Conception and design: RMP, JESS, LPC Analysis and interpretation: RMP, JESS, AOA, PRS, RDR, MHP, MVB, LPC Data collection: JESS, AOA, PRS, RDR, MHP Writing the article: RMP, JESS, AOA, PRS Critical revision of the article: RDR, MHP, MVB, LPC Final approval of the article*: RMP, JESS, AOA, PRS, RDR, MHP, MVB, LPC Statistical analysis: N/A Overall responsibility: RMP, JESS, MVB, LPC *All authors have read and approved of the final version of the article submitted to J Vasc Bras.
