# Erratum

**DOI:** 10.1590/1677-5449.001703

**Published:** 2018

 The article “Arterial bullet embolism after thoracic gunshot wound”, first published ahead of print on Aug 30, 2018 and finally in Jornal Vascular Brasileiro 17(3):262-266, DOI: https://doi.org/10.1590/1677-5449.005315 ; was published with the following sections missing, due to desktop publishing error:

- Correspondence,
- Author Information, and
- Author Contributions.

The missing content that should have been published may be found below:

**Correspondence**Leonardo Pessoa Cavalcante Hospital Universitário Francisca Mendes, Serviço de Cirurgia Vascular e Endovascular Av. Camapuã, 108 - Cidade Nova II CEP 69097-720 - Manaus (AM), Brazil Tel.: +55 (92) 3649-2750 / +55 (92) 3649-2839 E-mail: leonardocavalcante@usp.br 

**Author information**RMP - General surgeon, Programa de Residência Médica em Cirurgia Geral, Hospital Universitário Getúlio Vargas, Universidade Federal do Amazonas (UFAM). JESS - Vascular surgeon, Serviço de Cirurgia Vascular e Endovascular, Hospital Universitário Francisca Mendes, Universidade Federal do Amazonas (UFAM). AOA - Vascular surgeon, Programa de Residência Médica em Cirurgia Vascular, Hospital Universitário Getúlio Vargas, Hospital Universitário Francisca Mendes, Universidade Federal do Amazonas (UFAM); Graduate (Professional Master’s), Programa de Pós-graduação em Cirurgia, Faculdade de Medicina, Universidade Federal do Amazonas (UFAM). PRS - Vascular surgeon, Programa de Residência Médica em Cirurgia Vascular e Endovascular, Hospital Universitário Getúlio Vargas, Hospital Universitário Francisca Mendes, Universidade Federal do Amazonas (UFAM). RDR - Vascular surgeon, Serviço de Cirurgia Vascular, Hospital Universitário Francisca Mendes, Universidade Federal do Amazonas (UFAM). MHP - Vascular surgeon, Programa de Residência Médica em Cirurgia Vascular, Hospital Universitário Getúlio Vargas, Hospital Universitário Francisca Mendes, Universidade Federal do Amazonas (UFAM). MVB - Vascular surgeon, Serviço de Cirurgia Vascular e Endovascular, Hospital Universitário Francisca Mendes, Universidade Federal do Amazonas (UFAM); Professor, Faculdade de Medicina, Universidade Federal do Amazonas (UFAM); Graduate (Professional Master’s), Programa de Pós-graduação em Cirurgia, Faculdade de Medicina, Universidade Federal do Amazonas (UFAM). LPC - PhD in Sciences (Medicine), Universidade de São Paulo; Vascular surgeon, Serviço de Cirurgia Vascular e Endovascular, Hospital Universitário Francisca Mendes, Universidade Federal do Amazonas (UFAM); Professor, Programa de Pós-graduação em Cirurgia, Faculdade de Medicina, Universidade Federal do Amazonas (UFAM). 

**Author contributions**Conception and design: RMP, JESS, LPC Analysis and interpretation: RMP, JESS, AOA, PRS, RDR, MHP, MVB, LPC Data collection: JESS, AOA, PRS, RDR, MHP Writing the article: RMP, JESS, AOA, PRS Critical revision of the article: RDR, MHP, MVB, LPC Final approval of the article*: RMP, JESS, AOA, PRS, RDR, MHP, MVB, LPC Statistical analysis: N/A Overall responsibility: RMP, JESS, MVB, LPC *All authors have read and approved of the final version of the article submitted to J Vasc Bras. 

